# The Important Role of Leptin in Modulating the Risk of Dermatological Diseases

**DOI:** 10.3389/fimmu.2020.593564

**Published:** 2021-02-01

**Authors:** Xin Su, Ye Cheng, Dong Chang

**Affiliations:** Department of Cardiology, The Xiamen Cardiovascular Hospital of Xiamen University, Xiamen, China

**Keywords:** leptin, obesity, immune system, leptin receptor, dermatological diseases

## Abstract

It is an indisputable fact that obesity is associated with a series of health problems. One important hallmark of obesity is excessive accumulation of lipids in the adipocyte, especially triglyceride (TG). Currently, the adipocyte has been considered not only as a huge repository of excess energy in the form of fat but also as an important source of multiple hormones and cytokines called adipokines. In obesity, the adipocyte is dysfunctional with excessive production and secretion of pro-inflammatory adipokines, such as tumor necrosis factor α (TNF-α), interleukin 6 (IL-6), and leptin. On the other hand, accumulating evidence has shown that leptin plays a vital role in stimulating angiogenesis, controlling lipid metabolism, and modulating the production of pro-inflammatory cytokines. Furthermore, the various activities of leptin are related to the wide distribution of leptin receptors. Notably, it has been reported that enhanced leptin levels and dysfunction of the leptin signaling pathway can influence diverse skin diseases. Recently, several studies revealed the roles of leptin in wound healing, the hair cycle, and the pathogenic development of skin diseases, such as psoriasis, lupus erythematosus, and dermatological cancers. However, the exact mechanisms of leptin in modulating the dermatological diseases are still under investigation. Therefore, in the present review, we summarized the regulatory roles of leptin in the pathological progression of diverse diseases of skin and skin appendages. Furthermore, we also provided evidence to elucidate the complicated relationship between leptin and different dermatological diseases, such as systemic lupus erythematosus (SLE), psoriasis, hidradenitis suppurativa, and some skin tumors.

## Introduction

Obesity, defined as having a body mass index (BMI) greater than 30 kg/m^2^, is associated with numerous health problems that include the combination of insulin resistance, hypertension, and cardiovascular disease (CVD) (1). Worldwide, the prevalence of obesity has approximately doubled during the past several decades, resulting in multiple obesity-related pathological conditions and posing serious risks to future health (2).

Notably, one important hallmark of obesity is excessive accumulation of lipids in the adipocyte, especially triglyceride (TG). Currently, the adipocyte has been considered not only as a huge repository of excess energy in the form of fat but also as an important source of multiple hormones and cytokines, named adipokines (3). In obesity, the adipocyte is dysfunctional with excessive production and secretion of pro-inflammatory adipokines, such as tumor necrosis factor α (TNF-α), interleukin 6 (IL-6), and leptin (4). Accumulating evidence has shown the role of these adipokines in multiple pathophysiological processes, including energy homeostasis, lipid metabolism, immunological activity, and the development of dermatological diseases.

Leptin, a 16-kDa protein encoded by the obese gene on chromosome 7q31.3, is mainly synthesized and secreted by subcutaneous adipose tissue (5). As shown in previous studies, circulating levels of leptin are strongly associated with total body weight in mice, suggesting that alterations of leptin concentration play an important role in modulating the process of energy intake and lipid metabolism (6). On the other hand, due to a wide distribution of the leptin receptor, it is proposed that leptin possesses diverse functions which affect multiple biological processes in different tissues. Consistent with this notion, it has been shown that leptin plays a vital role in stimulating angiogenesis, controlling lipid metabolism, and modulating the production of pro-inflammatory cytokines (7).

It is worth noting that patients with skin diseases have a higher risk of subclinical metabolic syndrome and CVD compared to that in individuals without skin disease. Indeed, it is accepted that diverse skin diseases, such as psoriasis, lichen planus, connective tissue diseases, bullous diseases, vitiligo, and chronic urticarial diseases, are closely associated with cardio-metabolic disorders (8). However, the underlying mechanisms are still not elucidated. Concerning this notion, focus is recently shifting towards elucidating potential mechanisms whereby different adipokines regulate the pathological progression of skin diseases (9). Consistently, a vital role of leptin has begun to gain appreciation, but the exact function is still unclarified. According to recent studies, leptin influences dermatological pathophysiology and as a consequence, might have an important impact on skin diseases and other systemic autoimmune disorders. Therefore, in the present review, we summarized the vital role of leptin in the pathological progression of diverse diseases of skin and skin appendages. Furthermore, we also provided evidence to elucidate the complicated relationship between leptin and different skin diseases, such as systemic lupus erythematosus (SLE), psoriasis, hidradenitis suppurativa, and some skin tumors.

## Relationship Between Obesity and Diverse Skin Diseases

Taking into account the growing number of obese individuals and unquestionable participation of adiposity in many pathological processes, we could reasonably speculate that increased body fat mass may result in the disruption of normal processes in the skin. As reported, obesity is considered to be a status of chronic and low-grade inflammation which leads to the disturbed secretion of multiple cytokines, such as leptin, adiponectin, and chemokines, that play a modulatory role in inflammatory response (10). Actually, in the obese condition, the adipocyte is dysfunctional with aberrant production of pro-inflammatory adipokines, including tumor necrosis factor α (TNF-α), interleukin (IL)-6, leptin, visfatin, resistin, angiotensin II, and plasminogen activator inhibitor 1 (PAI-1). Noteworthy, the excessive pro-inflammatory adipokines within the circulation can presumably stimulate inflammatory signaling pathways and affect the development of obesity in turn. Thereby, leptin is currently regarded as the most important pro-inflammatory adipokine, since it stimulates the production of IL-1, IL-6, IL-12, and TNF-α by innate immune cells and enhances reactive oxygen species (ROS) production (11, 12).

On the other hand, systemic adiposity also results in alterations of dermatological physiology. Therefore, it has been proposed that obesity could be considered as one of the most vital risk factors for the development of several dermatological diseases (13). Indeed, obesity is confirmed to be associated with venous stasis, lymphedema, and an increased rate of infection, such as candidiasis, intertrigo, candida folliculitis, furunculosis, erysipelas, cellulitis, erythrasma, tinea cruris, folliculitis, and necrotizing fasciitis. Otherwise, the obese condition also increases the risk of selected inflammatory dermatoses, such as psoriasis, hidradenitis suppurativa, and atopic dermatitis (14).

Some dermatological abnormalities, such as acanthosis nigricans, keratosis pilaris, striae diseases, skin tags, and palmoplantar keratodermas, have been observed more commonly in obese patients than in those individuals with normal BMI (14, 15). With the development of obesity, elevated concentrations of hormones, including androgens, insulin, growth hormone, and insulin-like growth factor, could induce the escalation of sebum production which subsequently exacerbate acne (8). Therefore, according to published results, immune dysregulation and elevated levels of pro-inflammatory adipokines, in particular leptin, have a significant function in regulating skin biology and the risk of multiple dermatological diseases (16, 17).

## Potential Mechanisms of Leptin In Systemic Metabolism

Leptin is synthesized and secreted mainly by the white adipose tissue (WAT). However, a small amount of leptin is produced by the hypothalamus, pituitary, gastric mucosa, bone marrow, mammary epithelium, skeletal muscle, and placenta (7). Due to the technological advances, major breakthroughs have been made to explain biological function of leptin.

Leptin acts as a kind of pleiotropic hormone. Under obese or overweight conditions, the secretion of leptin is increased significantly. In addition, multiple other factors, such as insulin, glucose, estrogens, and diverse adipokines might also enhance the secretion of leptin (18). Interestingly, the peripheral leptin level is shown to follow a circadian rhythm with a peak at night. Furthermore, given that the serum leptin concentration is strongly correlated with the amount of body fat mass and the BMI value, some scholars have proposed that obesity should be characterized by enhanced circulating leptin levels (5). More recently, an independent research demonstrated that higher serum leptin concentrations coexisted with leptin resistance, and the disturbance was closely related to the development of obesity (19).

Aside from the established role of leptin in modulating the development of several dermatological diseases, the function of leptin receptors has also been given substantial attention in recent years. The expression of leptin receptor was found in the hypothalamus, fibroblasts, endothelial cells, keratinocytes, adipocytes, and blood mononuclear cells (20). After binding to its receptor in the hypothalamus, leptin activates an important signaling cascade which subsequently induces the inhibition of several orexigenic neuropeptides (21). Thus, we can speculate from these findings that the wide distribution of leptin receptors leads to the pleiotropic function of leptin.

Leptin receptor is a transmembrane receptor which is similar to the Class I cytokine receptors family (22). Owing to the differences within the basic structure, several isoforms of leptin receptor can be distinguished, including the short isoform and the full-length isoforms. To expand something in detail, the short isoforms of the leptin receptor comprise leptin receptor-A, leptin receptor-C, leptin receptor-D, and leptin receptor-F. The full-length isoform of leptin receptor contains leptin receptor-B (23). Notably, the full-length isoform of the leptin receptor is also considered to be responsible for controlling food intake and systemic energy homeostasis. Two short isoforms of leptin receptor, leptin receptor-A and leptin receptor-C, are located predominantly in micro-vessels within the central nervous system (CNS) where they are responsible for leptin circulation in the cerebrospinal fluid, as well as receptor-mediated transport of leptin through the blood-brain barrier (24). Additionally, leptin receptor-A and leptin receptor-C in extra-neural tissues have been verified to determine the functional pleiotropy of leptin; whereas the soluble isoform, leptin receptor-E, provides binding variety and bioavailability of leptin (25).

**Figure 1:**
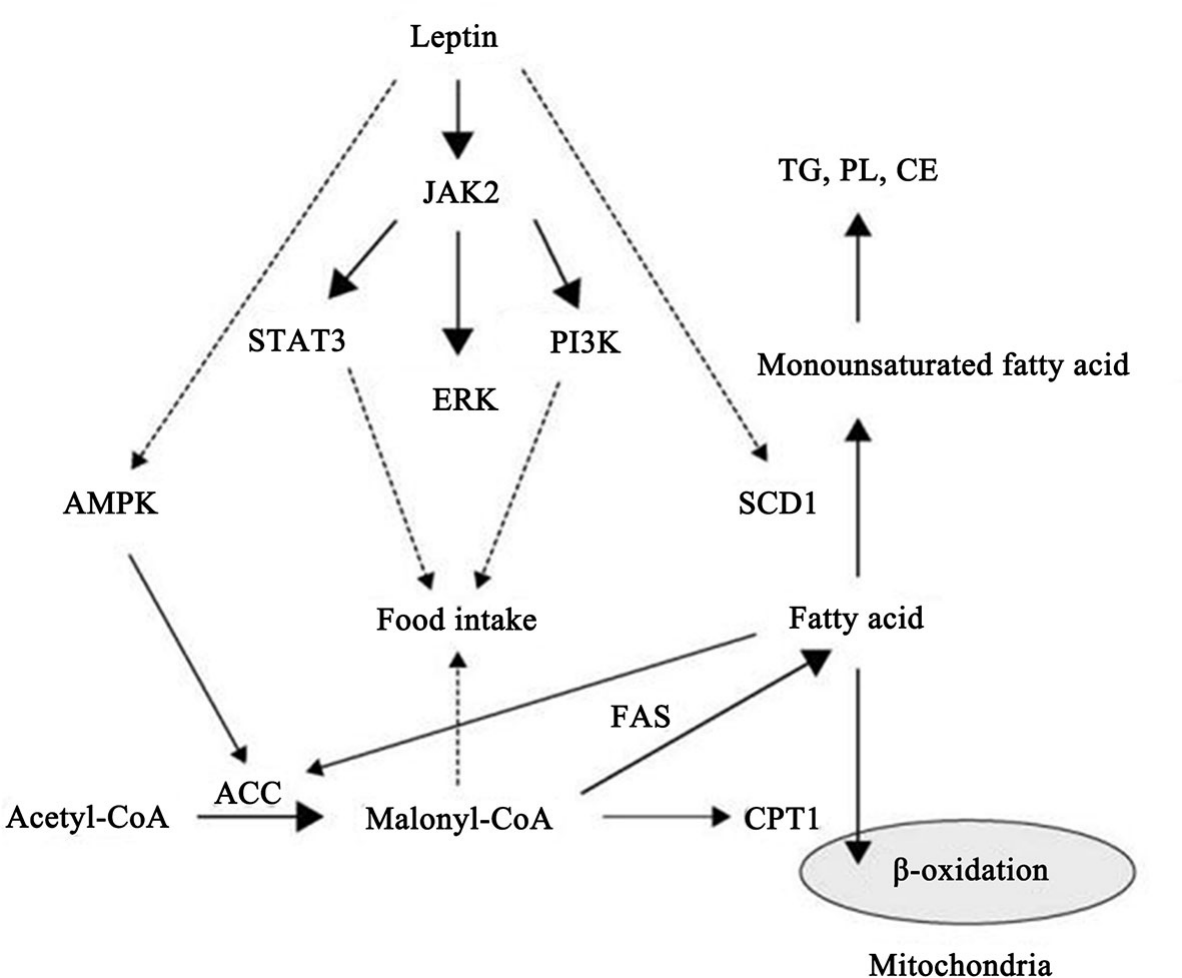
Schematic representation of leptin and leptin receptor signal transduction pathways and functions. Stimulation of the leptin receptor by leptin can activate JAK2 kinase, resulting in tyrosine phosphorylation of the receptor and downstream proteins, including STAT3, SHP2, IRS2, and PI3K, that play an important role in regulating transcription of genes essential for energy intake and lipid metabolism.

The important role of leptin in regulating intracellular signaling pathways has also begun to gain appreciation. According to published reports, leptin can stimulate multiple signaling pathways including the Janus kinase/signal transducer and activator of transcription (JAK/STAT) pathway, the phosphoinositide 3-kinase (PI3K) pathway, the mitogen-activated protein kinase (MAPK) pathway, the extracellular signaling-regulated kinase 1/2 (ERK1/2) pathway, the adenosine monophosphate kinase (AMPK) pathway, and the PPAR gamma coactivator/peroxisome proliferator-activated receptor (PGC/PPAR) pathway (26). Among these important signaling pathways, the JAK/STAT signal transduction cascade is the most important signaling pathway that can be activated by leptin. After binding of leptin with the full-length isoform of the leptin receptor, JAK is phosphorylated activated which subsequently promotes the phosphorylated stimulation of STAT3, leading to its dimerization followed by migration to the nucleus where STAT3 influences the expression of target genes, such as the suppressor of cytokine signaling 3 (SOCS3) (27). The comprehensive signaling and function of leptin and the leptin receptor are shown in **Figure 1**.

Recently, leptin has been found to play a role in modulating mitochondrial metabolism since it increases the efficiency of oxidation-reduction reactions and energy utilization within mitochondria (28). Taken together, the results summarized above could provide the potential mechanisms by which leptin and its receptor regulates the development of diverse skin diseases.

## Biological Functions of Leptin

Given that the biological functions of leptin are being extensively elucidated, the focus of this section is to discuss recent research elucidating the main functions of leptin. Under physiological condition, leptin exerts the ability to limit food intake, control body fat mass, and stimulate energy expenditure by negative feedback at the hypothalamic nuclei (29). However, in obesity status, the dysfunctional adipocyte may lead to an irrepressible increase in circulating leptin (30). This phenomenon might be a consequence of the dysfunction of leptin signaling pathways which may inhibit the combination of leptin with its receptors, followed by changes in leptin receptor expression or signal transduction (31).

**Figure 2:**
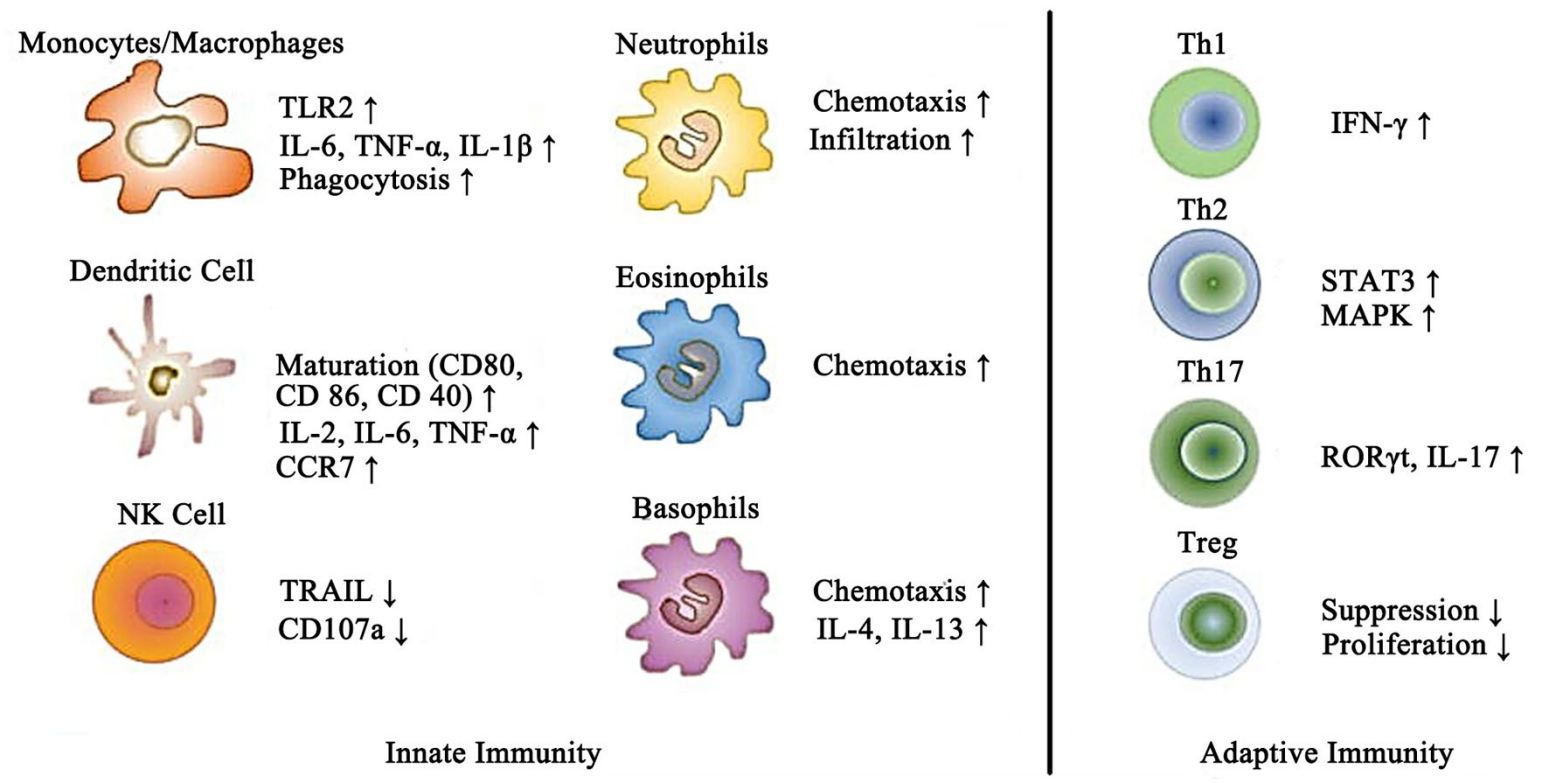
Schematic representation of the effects of leptin on different immune cells of the innate and adaptive immune systems.

On the other hand, it has been demonstrated that leptin also affects the immune system by acting as a pro-inflammatory adipokine. Indeed, as mentioned above, leptin itself activates the secretion of several other pro-inflammatory cytokines, as well as increases nitric oxide (NO) release and stimulates phagocytosis by monocytes/macrophages (32). Likewise, in neutrophils, leptin is also confirmed to induce the synthesis of oxygen free radicals, playing an essential role in the oxidative stress response. Recently, emerging evidence showed that leptin enhanced the cytotoxicity and proliferation of natural killer (NK) cells. Aside from the effects mentioned above, leptin also has the property to activate the chemotaxis of eosinophils, basophils, and neutrophils (33). It is noteworthy that leptin increases the production of several pro-inflammatory cytokines, such as IL-8, IL-12, IL-6, and TNF-α, from dendritic cells. Furthermore, by influencing lymphocyte receptors, leptin modulates the balance of Th1/Th2 lymphocytes towards the Th1 lymphocyte phentype, which subsequently leads to an aggravation of the inflammatory response. The wide impact of leptin on the immune system indicates the important role of leptin in the pathogenic development of immune diseases (34). The multiple effects of leptin on different types of immune cells of the innate and adaptive immune systems is shown in **Figure 2**.

## The Impact of Leptin On Hair And Diverse Skin Diseases

Although the adipocyte isolated from subcutaneous tissue is a prevalent site of leptin synthesis, it has been reported that fibroblasts and keratinocytes also possess the ability to produce leptin and express leptin receptors (35). With in-depth investigation, the expression of the leptin receptor has been detected in the epidermis, predominantly in the basal layer and the hair follicle papilla cells (36). Interestingly, results showed that leptin possessed the ability to stimulate the proliferation of keratinocytes and fibroblasts, as well as to facilitate epithelialization and collagen synthesis. These processes lead to an improvement in skin regeneration (37). Moreover, the local synthesis and secretion of leptin increases significantly after skin injury which results in shortening of the period of wound healing, suggesting an important role of leptin in skin regeneration (38). Additionally, independent research revealed that leptin also supported the skin micro-organism defense, which provided another potential mechanism whereby leptin regulated the wound healing process (39). However, by stimulating the STAT3 signaling pathway, leptin could trigger the proliferation, differentiation, and migration of diverse cell types in the skin, as well as modulating the pathological process of angiogenesis (40).

In 2018, Lee et al. conducted a research and demonstrated the molecular mechanism by which leptin modulated the proliferation of keratinocytes via examining the genome-wide transcriptional responses of normal human keratinocytes (NHKs) (35). In addition, leptin was found to enhance several intracellular signaling pathways, such as the PI3K signaling pathway, and induced a pro-inflammatory response in the keratinocyte. Enhanced function of leptin was induced by the increased production of interleukins in a similar mechanism as was observed within immune cells. Taken together, the above effects of leptin might have a significant impact on the risk and development of dermatological diseases and related chronic metabolic disorders (41, 42).

### Relationship Between Leptin and Hair

Owing to technological advances, several eye-catching breakthroughs have been put forward to further illuminate the association between leptin and hair. According to novel published results, immuno-histochemical analyses revealed the presence of leptin protein and leptin mRNA in different hair structures, including the matrix, the inner root sheath, and the follicular dermal papilla (43). Noticeably, in mice with leptin deficiency (the ob/ob mouse model), the period of the first anagen cycle was found to be delayed significantly. The authors suggested that leptin could be considered as an essential activator of anagen to promote the hair growth (43). Similarly, the injection of exogenous leptin stimulated anagen conversion in resting hair follicles (44). Nevertheless, the exact mechanism of the influence of leptin on the hair cycle and hair growth is still not fully elucidated (45). We still need further investigation to explore the relationship between leptin and the process of hair growth.

### Relationship Between Leptin and Psoriasis

Psoriasis is one of the most common skin diseases with a multifactorial pathogenesis. The inflammatory process underlying the skin changes usually has three features, including erythema, thickening, and scale. The typical psoriatic skin lesions present as silver-whitish scales with sharply demarcated, red, and thickened areas (46). According to the results of epidemiological investigation, the prevalence of psoriasis is various in different regions, but overall it reaches approximately 2% of the world population (47).

Recently, psoriasis has been considered to be a kind of genetic-, autoimmune-, and metabolic-derived disease. It has been speculated that when a genetically predisposed individual is exposed to specific environmental factors (EFs) that act along with epigenetic alternations, he/she may present psoriatic skin alterations (48, 49). Well-known EFs that are strongly linked to psoriasis include dietary habits along with obesity, microbiota, infections, alcohol intake, tobacco smoking, and psychological factors (50, 51).

There are numerous studies concerning serum adipokine levels during the course of psoriasis, and these observations comprise not only leptin but also resistin, adiponectin, and other inflammatory cytokines (52, 53). In addition, higher levels of leptin within the sera of psoriatic patients were observed in comparison with those in control participants (54, 55). On the other hand, remarkably increased leptin levels were found in obese individuals, particularly in those with obesity, suggesting a positive relationship between elevated levels of leptin and the risk of psoriasis and metabolic disorders (56). Interestingly, this finding was further replicated in another investigation conducted by Mitsuyama and colleagues. According to this report, the authors demonstrated that the expression of leptin mRNA was significantly enhanced in obese and psoriatic individuals compared to that in non-obese counterparts (57). Of note, some studies demonstrated robustly decreased levels of leptin in peripheral tissues after systemic therapy for psoriasis, such as using cyclosporine-A, suggesting a close relationship between serum levels of leptin and the pathological progression or regression of psoriasis (58).

Recently, a positive correlation between serum leptin concentration and the severity of psoriasis, which is evaluated by the Psoriasis Area and Severity Index (PASI), was also put forward (59). Using the ob/ob mice with imiquimod (IMQ)-induced psoriasis, Stjernholm et al. demonstrated that these mice exhibited attenuated manifestations of inflammation, including erythema, infiltration, and scaling in dorsal and ear skin (60). On the other hand, Shimoura et al. observed that IMQ-induced psoriatic-related inflammation was exacerbated in leptin receptor-deficient diabetic obese mice (db/db mice) compared with that in control mice, accompanied by significantly elevated expression of C-X-C motif ligand 5 (CXCL5) and IL-6. Importantly, massive number of CXCL5 positive cells infiltrated into the dermis and subcutaneous adipose tissue of the diabetic obese mice, suggesting a function of inflammatory cytokines in the increase of psoriasis severity with concomitant cardio-metabolic disorders (61). Due to its pro-inflammatory activity, leptin promoted production of IL-1, IL-6, and TNF-α, which also impacted the development and the severity of psoriasis (62). Notably, all the above-mentioned processes could stimulate the Th1/Th17 lymphocyte axis and induce a higher concentration of IL-17/IL-23 (63). Moreover, leptin is also confirmed to be involved in the activation of the Th1/Th17 lymphocyte axis via the JAK/STAT3 signaling pathway, which could further exaggerate angiogenesis in psoriatic lesions (64).

Recently, it has been suggested that enhanced levels of leptin in patient’s skin may induce the production of amphiregulin, which is an epidermal growth factor receptor ligand with a probable role in promoting keratinocyte proliferation (65). Furthermore, concentrations of CXCL8, which can also induce keratinocyte proliferation, were found to be increased remarkably in the skin of psoriatic patients (66). It is worth noting that leptin may also stimulate the production of CXCL8 by monocytes (67). According to these findings, we can reasonably speculate that leptin links obesity and psoriasis potentially via modulating the expression level of diverse chemokines within the circulation.

On the other hand, since alterations caused by mutation of the *LEPTIN* gene have been less addressed, the focus of the current paragraph is to discuss the recent work describing the relationship between single nucleotide polymorphisms (SNPs) of the *LEPTIN* gene and the development of psoriasis. For instance, the group of Torres et al. used the data from a Genome Wide Association Study (GWAS) and found that two important SNPs, including the *rs2167270* SNP and the *rs1137100* SNP, had no significant relationship with the development of arteriosclerosis and obesity in a cohort of psoriatic patients (68). Similar to these findings, results from research conducted by Karpouzis et al., who examined the *rs2060713* SNP, did not establish any link between the SNP and the severity of psoriasis (69). In contrast, Abdel Hay et al. observed that participants who carried the *G2548A* SNP presented elevated circulating levels of leptin as well as an increased risk of psoriasis, indicating that the *G2548A* SNP may be a novel predictor for the development of psoriasis (70).

However, more large-scale clinical investigations are still needed to further elucidate the comprehensive relationship between diverse SNPs of *LEPTIN* gene and the risk and severity of psoriasis.

### Relationship Between Leptin and Systemic Sclerosis

The lack of activation of the STAT3 signaling pathway has been shown to lead to multiple important alterations in the skin (71). Therefore, the question could be raised as to whether there is a link between insufficient activation of leptin signaling pathways and dermatological fibrotic diseases.

Several meta-analyses showed that in patients suffering from systemic sclerosis, serum leptin levels were comparable to those of healthy control individuals. For instance, to determine the relationship between serum leptin levels and disease activity, Budulgan et al. conducted a study enrolling 30 healthy individuals and 30 patients with systemic sclerosis. After analysis, the authors demonstrated that there was no significant difference between the two groups in terms of serum leptin levels. Nevertheless, the serum leptin levels were significantly reduced in patients with active systemic sclerosis, suggesting that leptin could be used as an activity biomarker in patients with this disease (72). Likewise, these findings were replicated in another study conducted by Olewicz-Gawlik and colleagues. According to these results, the authors also found no statistically significant difference in circulating levels of leptin between patients with systemic sclerosis and healthy control individuals. However, circulating concentrations of leptin were shown to be correlated with the duration of symptoms (73). A meta-analysis containing 14 studies also showed similar results (74). Thus, it seems that serum levels of leptin are not significantly different between patients with systemic sclerosis and healthy individuals.

On the contrary, several studies showed that the serum levels of leptin increased in patients with systemic sclerosis. In 2012, Pehlivan et al. showed that circulating leptin levels were significantly increased by approximately 5-fold in patients with systemic sclerosis compared with those in healthy controls (75). More recently, another study which enrolled 100 patients with confirmed systemic sclerosis diagnosis and 20 healthy individuals revealed that the patients presented significantly elevated serum levels of leptin (76). The discordant results summarized above might be attributed to diverse age, sex, and race of the enrolled participants. Further studies, including large-scale study of many subjects, should be carried out to clarify this relationship.

The underlying mechanism whereby leptin facilitates the development of systemic sclerosis could be also attributed to the function of leptin in indirectly stimulating the development of fibrosis. It has been well demonstrated that binding of leptin to its receptor in regulatory T lymphocytes is a signal for decreased proliferation of regulatory T lymphocytes which could enhance fibrosis (77). In addition, leptin has also been suggested to promote fibrosis by an alternative mechanism related to the renin–angiotensin system (RAS). What is more, in the elderly population, leptin is shown to be associated with arterial stiffness (78), which is one of the features of vascular fibrosis. Accordingly, leptin might be useful as a potential marker of vascular damage in systemic sclerosis.

### Relationship Between Leptin and Systemic Lupus Erythematosus

Systemic lupus erythematosus (SLE) is characterized by excessive accumulation of anti-nuclear auto-antibodies, hyper-activation of immune cells, and aberrant infiltration of T lymphocytes, leading to the systemic damages triggered by immune complexes deposition (79). Importantly, with more advanced disease, several other organs and systems, including the renal, pulmonary, cardiovascular, and central nervous systems, are also affected. In terms of the responses of immune cells, the defective erasure of auto-reactive lymphocytes, the immune responses against autoantigens, and the loss of self-tolerance, are also vital contributing factors to susceptibility of SLE.

It has already been confirmed that serum levels of leptin increased significantly in patients with SLE, a finding that has been replicated in many studies from diverse countries, revealing that leptin can be considered as a novel biomarker in predicting the risk of SLE (80–83). Recently, several possible mechanisms by which leptin modulates the development of SLE have been emphasized. For instance, using mouse models, it was shown that leptin promoted the differentiation of Th17 lymphocytes and enhanced synthesis of IL-17 via interacting with the RAR-related orphan receptor gamma (ROR-γ) (84). Moreover, leptin could function together with another pro-inflammatory factor, neutrophil-activating protein (NAP-2), and activate the PI3K/Akt signaling pathway in SLE patients (85). On the other hand, leptin promoted survival and proliferation of auto-reactive T lymphocytes in mice with an SLE-like mutation (86). An inhibiting effect of leptin on T regulatory lymphocytes was also suggested (77).

Leptin could influence immune cell subsets, modulate cytokine secretion, and promote anti-apoptotic protein expression. Nevertheless, the interaction between leptin and other inflammatory cells under SLE condition is not fully understood (87). Additionally, no significant difference was observed between patients with active and those with inactive SLE (80, 88, 89). Nevertheless, a lower leptin concentration was found with joint inflammation and neurological symptoms during a course of SLE (90). More studies, including large-scale clinical trials and animal research, are still needed to clarify the relationship between leptin and SLE and the underlying mechanisms.

### Relationship Between Leptin and Skin Tumors

As described in previous studies, in obesity, dysfunctional adipocytes expressed excessive leptin which subsequently increased the risk of many skin tumors (91–93). Recently, it was also demonstrated that an increased leptin concentration may accelerate the growth of melanoma, to increase mortality in patients (94, 95). Recent research has also shown a positive correlation between serum leptin levels and the number of skin tags. Furthermore, either the higher circulating levels of leptin or the reduced levels of leptin receptors can trigger the proliferation and differentiation of keratinocytes into skin tag lesions (96, 97). Taken together, these findings highlight the function of leptin in promoting the development of dermatological tumors.

### Relationship Between Leptin and Hidradenitis Suppurativa

According to the latest results, an important relationship between leptin and hidradenitis suppurativa has begun to gain appreciation since leptin plays an important role in pro-inflammatory responses. To expand, it has been found that obesity is an independent risk factor for hidradenitis suppurativa, and the risk for patients with hidradenitis suppurativa to suffer from obesity and metabolic syndrome is relatively higher compared to that in healthy controls. Consistent with this notion, Malara and colleagues observed that serum levels of leptin were significantly increased in patients with hidradenitis suppurativa (98). On the other hand, it should be emphasized that enhanced local concentrations of leptin in subcutaneous adipose tissue could cause an intensification of the inflammatory response within the skin of patients with hidradenitis suppurativa. This marked alteration may subsequently result in an increase of systemic inflammation and exacerbate the symptoms of hidradenitis suppurativa (99). The potential role of leptin in selected skin diseases is summarized and presented in **Table 1**.

**Table 1:** The potential role of leptin in diverse skin diseases.

Skin diseases	Serum level of leptin	Potential effect	References
Psoriasis	Elevated	Pro-inflammatory: promotion of Th1/Th17 lymphocyte axis (IL-1↑, IL-6↑, IL-17/23↑, CXCL8↑, TNF-α↑); Angiogenesis: activating the JAK/STAT3 signaling pathway; Keratinocyte proliferation: amphiregulin↑, CXCL8↑	(55–69)
Systemic sclerosis	Not significantly changed/Elevated	Anti-fibrotic: inhibiting the JAK/STAT3 signaling pathway	(71–73) (74–75)
Systemic lupus erythematosus	Elevated	Pro-inflammatory: promotion of the Th1/Th17 lymphocyte axis, inhibition of Treg lymphocytes; Aging of mesenchymal stem cells (via the NAP-2 and PI3K-Akt pathway)	(79–86)
Melanoma and non-pigment tumors	Elevated	Angiogenesis: JAK/STAT3 pathway↑, VEGF↑; Mitogenic	(90–94)
Skin tags	Elevated	Promotion of keratinocyte and fibroblast growth	(95, 96)
Hidradenitis suppurativa	Elevated	Pro-inflammatory: promotion of Th1 lymphocytes	(97, 98)

## Conclusions and Perspectives

Leptin is a pluripotent adipokine and is strongly correlated with multiple immune responses in humans. The pleiotropic effects of leptin in modulating the pathogenic development of various skin diseases have been well demonstrated. As a consequence of adiposity and increased leptin levels, especially in conjunction with reduced expression of leptin receptors, several pathological processes have been observed in the skin and the skin appendages. Although the potential mechanisms whereby leptin modulates disease development have been thoroughly investigated, the exact role of leptin in dermatological disorders still needs to be further elucidated.

On the other hand, it is a challenge to explore all of the potential confounding factors which affect dermatological pathology. Moreover, it is also important to further find out the exact mechanism whereby those factors affect skin and skin appendages. It needs to be highlighted that not all activities of leptin in the skin are fully understood. Another unsolved problem is that there is no clear explanation of why some obese individuals present with a worse course of skin diseases. It is worth noting that the recently postulated concept for leptin, “the less leptin is more” in the obese setting, may offer a better explanation for this issue. Otherwise, it is still an open question of whether pharmacological intervention resulting in decreased leptin secretion or breaking the leptin resistance could be a target for the treatment of different skin diseases. Actually, several leptin antagonists and leptin-neutralizing antibodies have been developed and could provide possible therapies for treating leptin-related dermatological diseases.

## Author Contributions

XS and DC contributed to the study design. XS wrote the manuscript. All authors contributed to the article and approved the submitted version.

## Funding

This work was supported by grants from the National Key Research and Development Program of China (no. 2016YFC1301202).

## Conflict of Interest

The authors declare that the research was conducted in the absence of any commercial or financial relationships that could be construed as a potential conflict of interest.
